# Diagnostic value of chest radiography in the early management of severely injured patients with mediastinal vascular injury

**DOI:** 10.1007/s00068-022-01966-3

**Published:** 2022-04-07

**Authors:** Christopher Spering, Soehren Dirk Brauns, Rolf Lefering, Bertil Bouillon, Corinna Carla Dobroniak, László Füzesi, Mark-Tilmann Seitz, Katharina Jaeckle, Klaus Dresing, Wolfgang Lehmann, Stephan Frosch

**Affiliations:** 1 Department of Trauma Surgery, Orthopedics and Plastic Surgery, Göttingen University Medical Center, Göttingen, Germany; 2 Department of Orthopedics and Traumatology, Spitalregion Rheintal Werdenberg Sarganserland, Rebstein, Switzerland; 3 Institute for Research in Operative Medicine (IFOM), University of Witten/Herdecke, Cologne, Germany; 4 Department of Trauma Surgery, Orthopedics and Sports Traumatology, Cologne, University of Witten/Herdecke, Cologne, Germany; 5 Pathology, Faculty of Medicine, University of Augsburg, Augsburg, Germany

**Keywords:** Mediastinal vascular injury, Early diagnostic severely injured patients, Mediastinal/chest ratio, Diagnostic in trauma resuscitation unit, Management of severely injured patients

## Abstract

**Introduction:**

Time is of the essence in the management of severely injured patients. This is especially true in patients with mediastinal vascular injury (MVI). This rare, yet life threatening injury needs early detection and immediate decision making. According to the ATLS guidelines [American College of Surgeon Committee on Trauma in Advanced Trauma Life Support (ATLS^®^), 10th edn, 2018], chest radiography (CXR) is one of the first-line imaging examinations in the Trauma Resuscitation Unit (TRU), especially in patients with MVI. Yet thorough interpretation and the competence of identifying pathological findings are essential for accurate diagnosis and drawing appropriate conclusion for further management. The present study evaluates the role of CXR in detecting MVI in the early management of severely injured patients.

**Method:**

We addressed the question in two ways. (1) We performed a retrospective, observational, single-center study and included all primary blunt trauma patients over a period of 2 years that had been admitted to the TRU of a Level-I Trauma Center. Mediastinal/chest (M/C) ratio measurements were calculated from CXRs at three different levels of the mediastinum to identify MVI. Two groups were built: with MVI (VThx) and without MVI (control). The accuracy of the CXR findings were compared with the results of whole-body computed tomography scans (WBCT). (2) We performed another retrospective study and evaluated the usage of sonography, CXR and WBCT over 15 years (2005–2019) in level-I–III Trauma Centers in Germany as documented in the TraumaRegister DGU^®^ (TR-DGU).

**Results:**

Study I showed that in 2 years 267 patients suffered from a significant blunt thoracic trauma (AIS ≥ 3) and met the inclusion criteria. 27 (10%) of them suffered MVI (VThx). Through the initial CXR in a supine position, MVI was detected in 56–92.6% at aortic arch level and in 44.4–100% at valve level, depending on different M/C-ratios (2.0–3.0). The specificity at different thresholds of M/C ratio was 63.3–2.9% at aortic arch level and 52.9–0.4% at valve level. The ROC curve showed a statistically random process. No significant differences of the cardiac silhouette were observed between VThx and Control (mean cardiac width was 136.5 mm, *p* = 0.44). Study II included 251,095 patients from the TR-DGU. A continuous reduction of the usage of CXR in the TRU could be observed from 75% in 2005 to 25% in 2019. WBCT usage increased from 35% in 2005 to 80% in 2019. This development was observed in all trauma centers independently from their designated level of care.

**Conclusion:**

According to the TRU management guidelines (American College of Surgeon Committee on Trauma in Advanced Trauma Life Support (ATLS®), 10th edn, 2018; Reissig and Kroegel in Eur J Radiol 53:463–470, 2005) CXR in supine position is performed to detect pneumothorax, hemothorax and MVI. Our study showed that sensitivity and specificity of CXR in detecting MVI was statistically and clinically not reliable. Previous studies have already shown that CXR is inferior to sonography in detecting pneumothorax and hemothorax. Therefore, we challenge the guidelines and suggest that the use of CXR in the early management of severely injured patients should be individualized. If sonography and WBCT are available and reasonable, CXR is unnecessary and time consuming. The clinical reality reflected in the usage of CXR and WBCT over time, as documented in the TR-DGU, seems to support our statement.

## Introduction

According to present Trauma Resuscitation Unit (TRU) management guidelines chest radiography (CXR) still remains one of the first-line imaging examinations of the thorax in the early management of severely injured patients to detect hemothorax, pneumothorax or mediastinal vascular injury (MVI) [3, 22]. CRX also is easily accessible and available in all TRUs independently from the designated trauma level. Yet thorough interpretation is essential for accurate diagnosis and drawing appropriate conclusion for further management. Thus it can render additional diagnostic steps unnecessary. Time and highly efficient management are of the essence in severely injured patients especially with MVI. Complete assessment of the pattern of injury is important for rapid team decision within the TRU. When patients are in critical condition, CXR may be the only imaging examination next to extended focused assessment sonography in trauma (eFAST) that can feasibly be performed without risking further injury or decompensation. Present studies have ascribed superior accuracy in detecting pneumothorax and hemothorax to eFAST [7, 12], but not for detecting MVI. While WBCT being increasingly easy accessible and defining the golden standard in diagnostic value, it needs to be available and reasonable to transfer the patient.

CXR taken in trauma patients within the TRU are not able to meet the standard imaging criteria due to imaging in the supine position on a stretcher. This fact can complicate injury diagnostics and lead to magnification effects. These effects can cause the perception of pseudocardiomegaly and mediastinal widening [13]. Taking these limitations into account, CXR still can provide the trauma team with a wide spectrum of valuable information, if the physicians are capable in diagnosing the radiological signs. [13].

The manifestations of severe thoracic injury in polytrauma are diverse, depending on both the mechanism of injury and the organ system or systems affected. Especially blunt chest trauma leading to MVI still ranks among the most serious clinical problems due to the difficulties of initial diagnostics performed in the TRU combined with a dramatically high mortality rate within the first hour after injury [10, 27]. While MVI is a rare event, at the same time, blunt chest trauma with an Abbreviated Injury Scale (AIS) > 2 is one of the leading diagnoses in severely injured patients [4, 10]. The initial management of these patients is a critical period, combining the urge of a thorough injury assessment with finding the sources of instability, stabilizing vital functions, and defining a therapeutic strategy. An easily accessible and highly sensitive diagnostic tool to disprove the presence of a mediastinal injury is crucial. Thus, the Committee on Trauma of the American College of Surgeons retains CXR in the 10th edition of its Advanced Trauma Life Support (ATLS) [3]. Several radiographic findings in CXRs have been evaluated in the past century to detect aortic rupture or mediastinal bleeding such as mediastinal widening, abnormal aortic contour, rib and other bone fractures, pneumothorax, hemothorax, and pulmonary contusion [8, 13, 17, 18, 27]. Nevertheless, the role of CXR in detecting pneumo- and hemothorax has been challenged in the past decade [7, 12, 15]. The eFAST is the superior diagnostic modality in initial trauma management [1, 2, 7, 8, 15, 24]. The combination of eFAST and the clinical evaluation of the patient can safely direct them towards immediate whole-body computed tomography (WBCT) without further diagnostics such as CXR [14]. While the diagnostic performance of eFAST is convincing in the context of early trauma management [1, 2, 8, 15, 20, 24]. CXR is performed in the supine position with limited diagnostic value, but can potentially provide much more information than eFAST, especially in the context of MVI [20]. Therefore, the diagnostic imaging in the early trauma management needs to be reevaluated.

This article discusses the utility of CXR in the early management of severely injured patients with MVI. Furthermore, we investigated which role CXR, WBCT and sonography are playing in the reality of daily routine within the early trauma management shown in the TR-DGU.

## Methods

The present retrospective study was performed at the Department of Trauma Surgery, Orthopedics and Plastic Surgery of University Medicine Göttingen. It includes all patients with primary blunt chest trauma who had been admitted to the TRU and CXR had been performed within a time period of 24 months. All patients underwent initial physical examinations and plain film CXR before a WBCT was performed.

The study was approved by the ethics committee at University Medical Centre Göttingen (DOK_121-2016), while informed consent for the use of data of all participating patients existed. All methods were performed in accordance with the guidelines and regulations of the German ethics committee as well as the General Data Protection Regulation of the European Union.

The University Hospital of Göttingen is a Level-I Trauma Center with an average of 900 severely injured trauma patients admitted to the TRU each year. All patients admitted to the TRU are suspected to be severely injured, according to the regional triage system [29]. The standard trauma care at the hospital is in concordance with the Advanced Trauma Life Support (ATLS) protocol and in accordance to the German Trauma Society (2016) [3, 22].

**Fig. 1:**
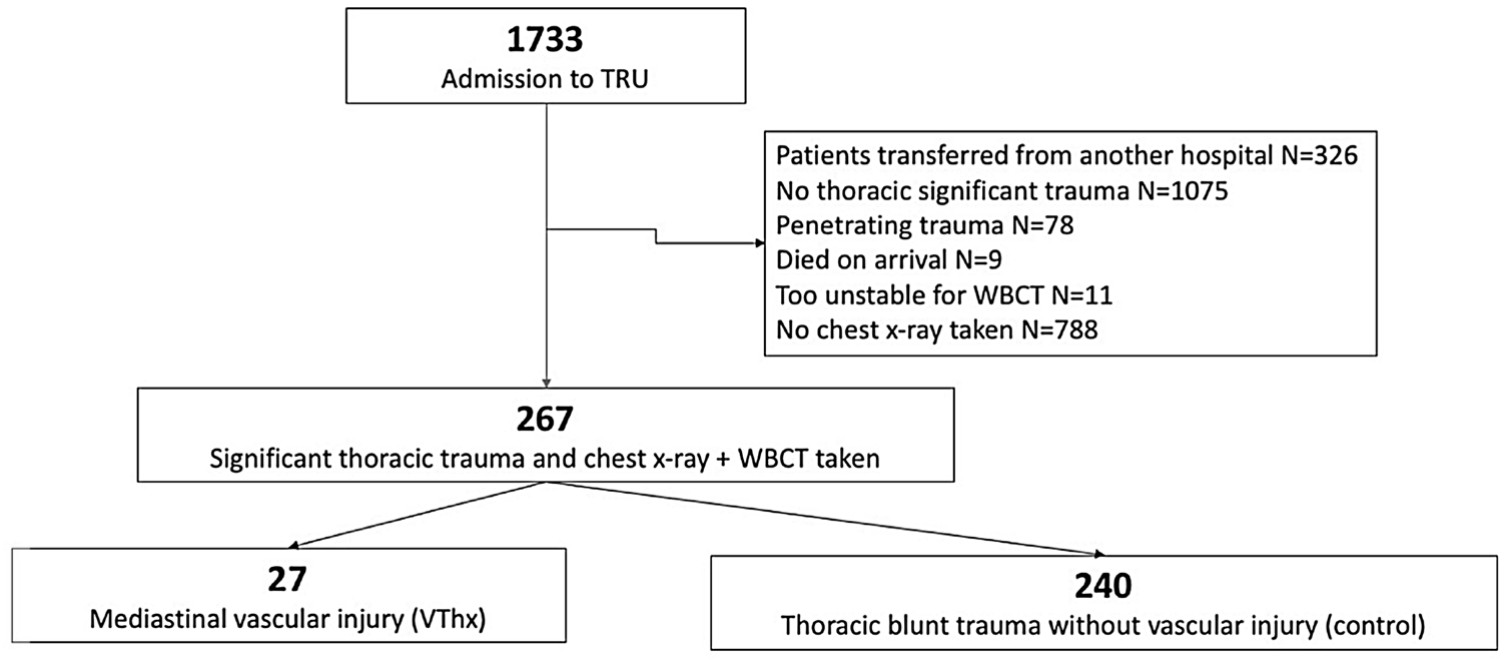
Flow chart of the study group. (WBCT whole-body computed tomography scan)

All CXR that had been taken during TRU management of the patients where retrospectively examined. After review of quality and diagnostic matters, three measurement levels were obtained to calculate the mediastinal/chest (M/C) ratio (Fig. 1):

- the width of the mediastinum at the aortic arch (Aʹ) and the width of the chest at the same level (Bʹ);
- the width of the mediastinum at the valve level (A) and the width of the chest at the same level (B); and
- the width of the cardiac silhouette (a) and the width of the chest at the same level (b).

The CXR was especially assessed for MVI and compared with the WBCT findings.

Two populations were identified when matching the patients’ data with the WBCT findings: those with MVI (VThx, *N* = 27; including aortic injury, pulmonary vessel and caval vein injuries) and those with significant blunt thoracic injury without MVI (control, *N* = 240). Further outcome parameters in correspondence of the presence of a vascular injury were assessed.

To identify the usage of CXR over time within German trauma centers, data have been acquired from the TR-DGU [30]. Primary admitted patients with serious injuries (need for intensive care) documented in the years 2005–2019 were analysed regarding diagnostic procedures in the emergency room. Access to these data have been approved by the AUC—Academy for Trauma Surgery (AUC, Munich).

After data anonymization and data bank acquisition, statistics were performed using an Excel spreadsheet (Microsoft Excel for Mac 2011; V. 14.3.4; Microsoft Redmond, Washington), SPSS (V. 23.0.0; IBM SPSS Statistics SPSS Inc., Chicago, Illinois), and Statistica (V. 12.7; StatSoft, Tulsa, Oklahoma). Data were categorized into nominal, metric, and ordinal levels. For nominal levels, the following tests were applied to show significance: Fisher exact test, Pearson Chi–squared test, and M–L Chi–squared test. For the metric level, *t* test or Mann–Whitney *U* test was applied, while for ordinal data only the Mann–Whitney *U* test was applied. After the first analysis, significance was considered as a *p* value of < 0.05, and the Bonferroni method was used afterwards, resulting in a *p* value of 0.002381.

Receiver operating characteristic curve (ROC curve) were calculated with the Youden Index to identify optimal sensitivity and specificity in different M/C ratios.

## Results

1733 patients were admitted to the TRU with complete recording during the study period, including 658 blunt thoracic trauma patients. Of these, 267 showed significant thoracic injury (AIS ≥ 3) and fulfilled the inclusion criteria (Fig. 1). 27 patients (10%) suffered MVI (VThx). The demographic and clinical characteristics of the patients are listed in Table 1, showing no difference in mean age and gender between VThx and Control. The mechanism of injury was different showing a larger rate of high velocity accidents in the VThx group. The Injury Severity Score (ISS) was different between groups (24 vs. 48 points) and so was the mortality rate (18.2% vs. 8.4%).

**Table 1:** Demographic and clinical characteristics of patients with mediastinal vascular injury (MVI) VThx compared to patients without MVI Control

	Patients with MVI (VTHX) * N* = 27	Thoracic injury without MVI (Control) * N* = 240
Mean age (years)	45	50
Males (%)	85	76
ISS (points)	24	48
Mechanism of injury		
Road traffic accident (%)	52	67
Fall > 3 m height	40	26
Others	8	7
Mortality (%)	18.2	8.4

**Fig. 2:**
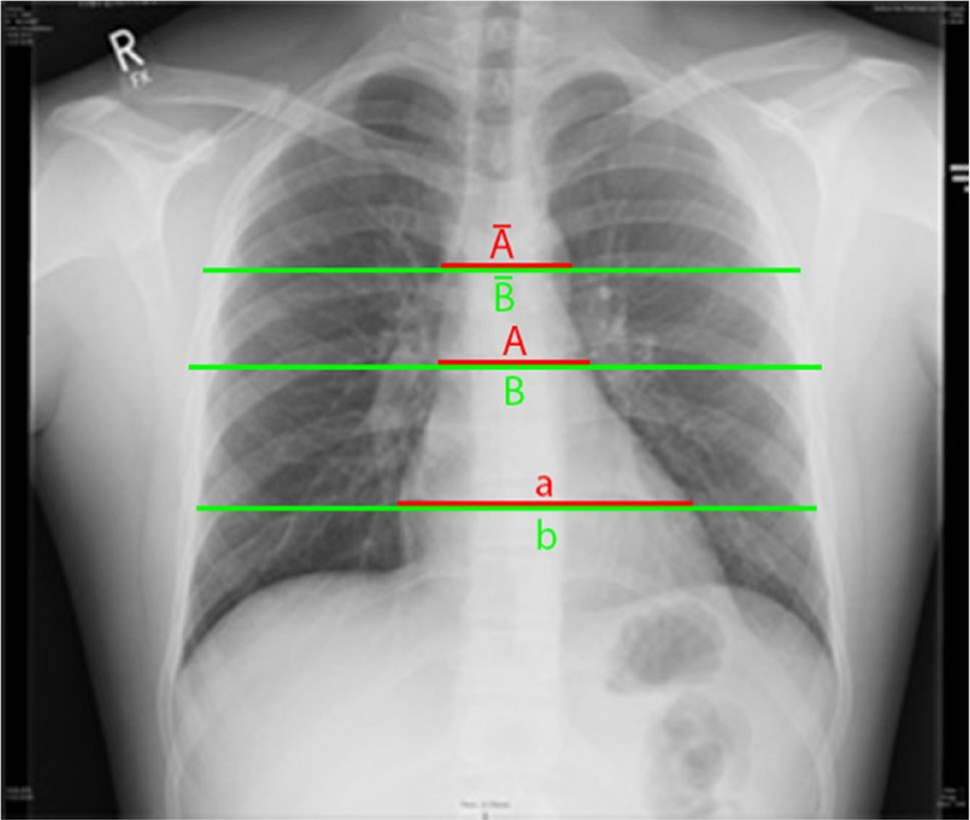
Measurement of mediastinal to chest width (M/C ratio) at the level of the aortic arch (*A*ʹ/*B*ʹ), valve level (*A*/*B*), and cardiac silhouette (*a*/*b*)

**Fig. 3:**
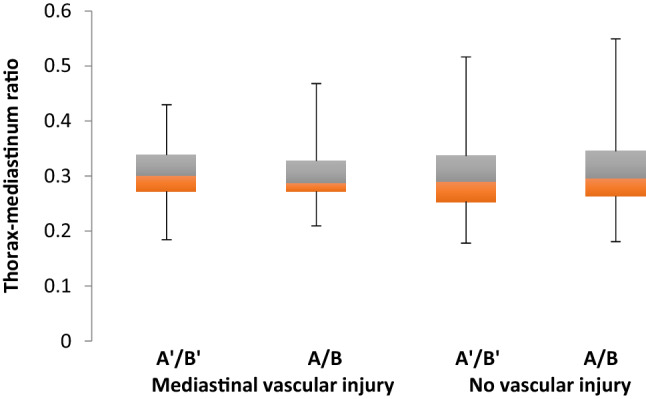
Comparison of mean M/C-ratios of VThx (*N* = 27) and Control (*N* = 240) at the aortic arch (*A*ʹ/*B*ʹ) vs. valve level (*A*/*B*)

To detect a MVI the measurements of the M/C-ratios at the aortic arch, valve level and cardiac silhouette were compared between VThx and control (Fig. 2). Patients with MVI (VThx) showed no significant difference of the M/C ratio compared to the control group. In the measurement at valve level (*A*/*B*), the ratio showed a median of 0.3 in both groups. In the measurement at the aortic arch level (*A*ʹ/*B*ʹ), the ratio was slightly higher in VThx (0.3 vs. 0.29) (Fig. 3).

**Fig. 4:**
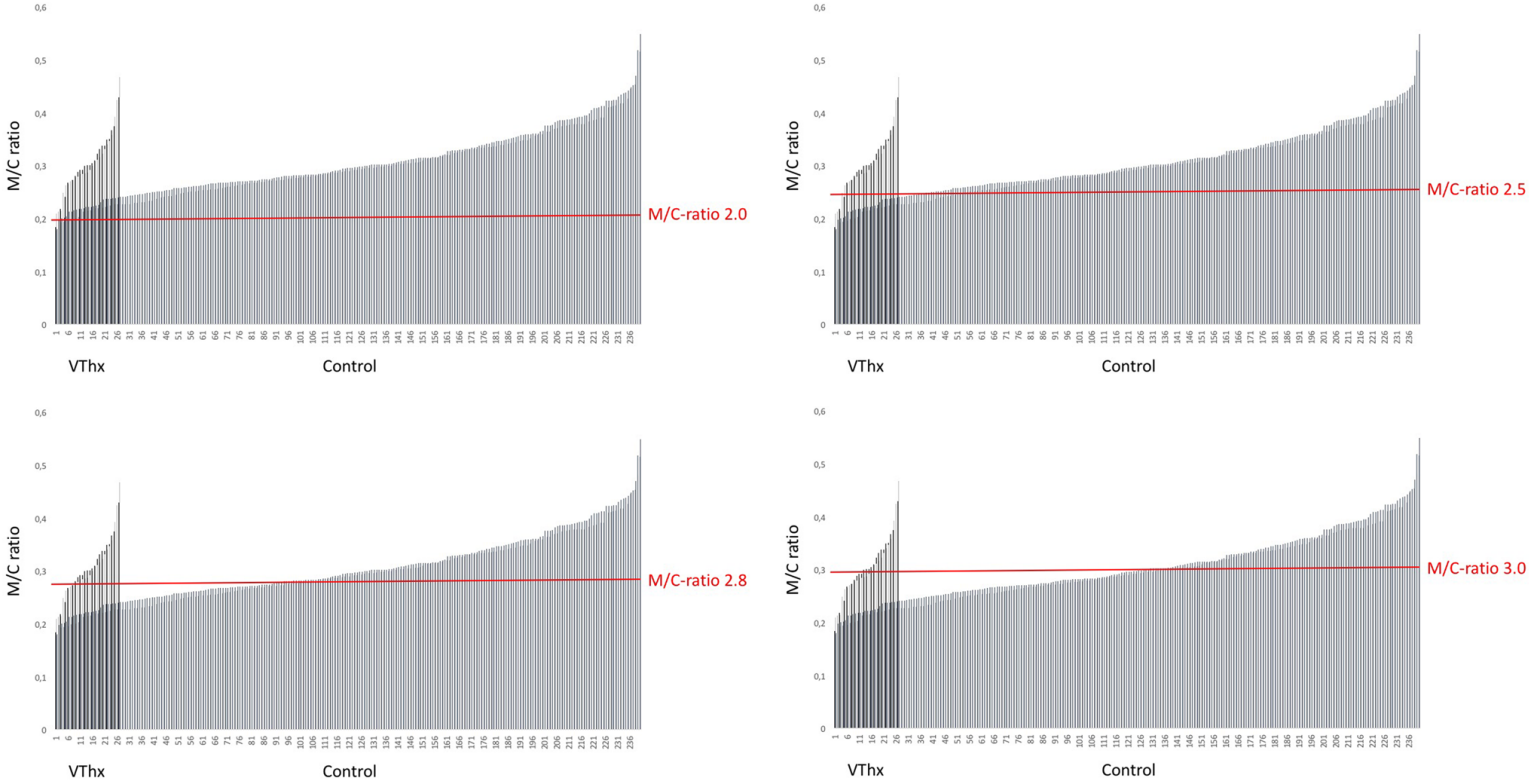
All M/C-ratios at measure points aortic arch (*A*ʹ/*B*ʹ) and valve level (*A*/*B*) in VThx versus Control, with threshold M/C-ratios of 2.0, 2.5, 2.8, and 3.0

**Fig. 5:**
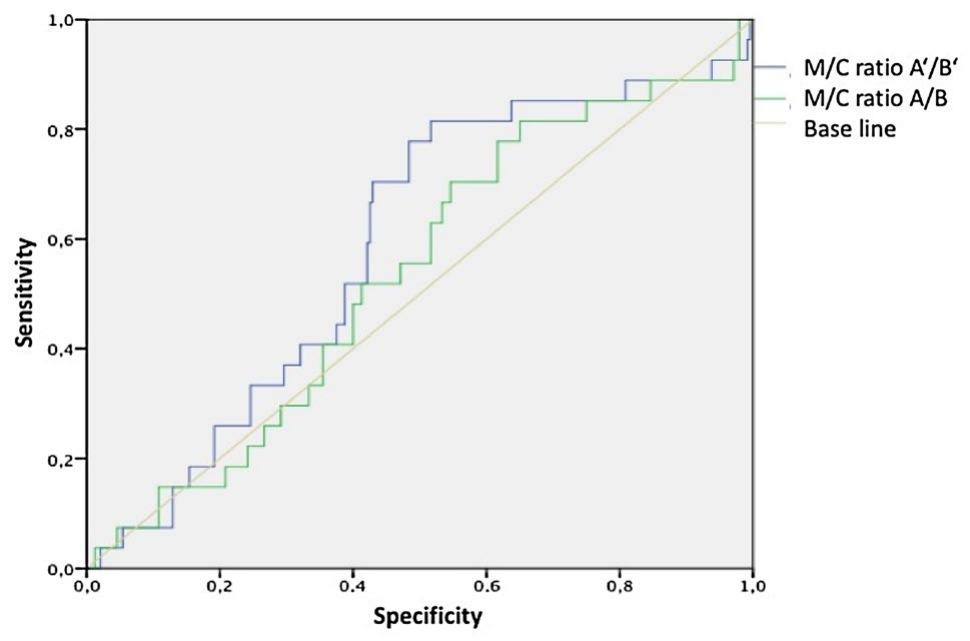
Receiver operating characteristic (ROC) curve after Youden Index showing a statistically random process, identifying a theoretical optimal sensitivity and specificity at M/C ratio *A*ʹ/*B*ʹ 0.3 and at *A*/*B* 0.28

In concordance to general suggestions [27] that a M/C ratio at the aortic arch > 0.25 is highly likely to be indicative of an aortic rupture in trauma patients, the data were tested concerning specificity and sensitivity. If a M/C ratio of 0.2 was considered pathological, a pathological result was diagnosed in 97% of the patients. This equates to a false positive finding in 233 patients who did not suffer from a MVI, and a specificity of 2.9%. At a M/C ratio of 0.25, it would still have been 78% positive with a specificity of 23% and sensitivity of 82%. At a M/C ratio of 0.28, the result was still unsatisfactory, leading statistically to a ratio of 0.3 with the best ratio regarding sensitivity to specificity (Table 2). At the mediastinal valve level a ratio of 0.28 showed the statistically best ratio of sensitivity to specificity (Table 3). A clinically acceptable sensitivities or specificities have been missed in all possible ratios (Figs. 3, 4, 5).

**Table 2:** Different mediastinal/chest ratios (M/C ratio) at the aortic arch level (*A*ʹ/*B*ʹ)

Ratio	≥ 0.2	≥ 0.25	≥ 0.28	≥ 0.3
Sensitivity (%)	92.6	81.5	70.4	56
Specificity (%)	2.9	22.5	43.3	63.3
Positive predictive value (%)	9.7	10.6	12.3	14.6
Negative predictive value (%)	77.8	91.5	92.9	92.7

**Table 3:** Different mediastinal/chest ratios (M/C ratio) at the valve level (*A*/*B*)

Ratio	≥ 0.2	≥ 0.25	≥ 0.28	≥ 0.3
Sensitivity (%)	100	85.2	63	44.4
Specificity (%)	0.4	16.7	38.3	52.9
Positive predictive value (%)	10.2	10.3	10.3	9.6
Negative predictive value (%)	100	90.9	90.2	89.4

After having performed the Youden Index and calculated the receiver operating characteristic curve (ROC) to identify the optimal ratio of sensitivity to specificity (Figs. 4, 5) (see ratios also shown in red in Tables 2, 3), 0.3 was identified for the mediastinal measurement at aortic arch level (*A*ʹ/*B*ʹ) and 0.28 for mediastinal measurement at valve level (*A*/*B*), which still missed about 44% of the MVI. Thus the ROC curve postulates a statistically random process.

The measurement of the cardiac silhouette showed a mean cardiac width of 136.5 mm, ranging from 84.6 to 216.5 mm, with no significant differences between VThx and control (*p* = 0.44 after Fischer exact test).

**Fig. 6:**
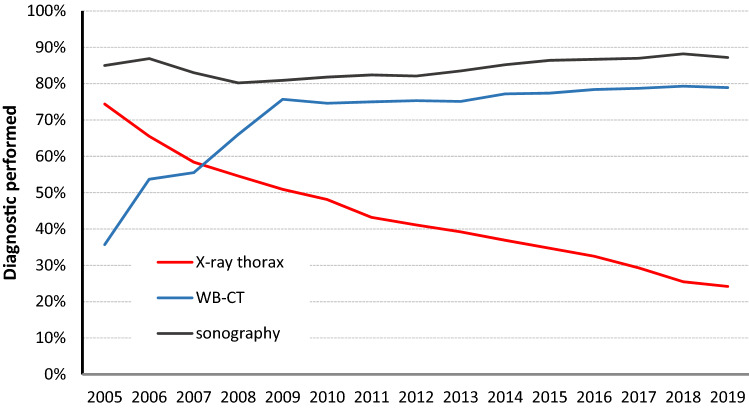
Frequency of different diagnostic procedures in severely injured trauma patients over time (data from TR-DGU, Germany 2005–2019, *n* = 251,095 primary admitted patients)

**Fig. 7:**
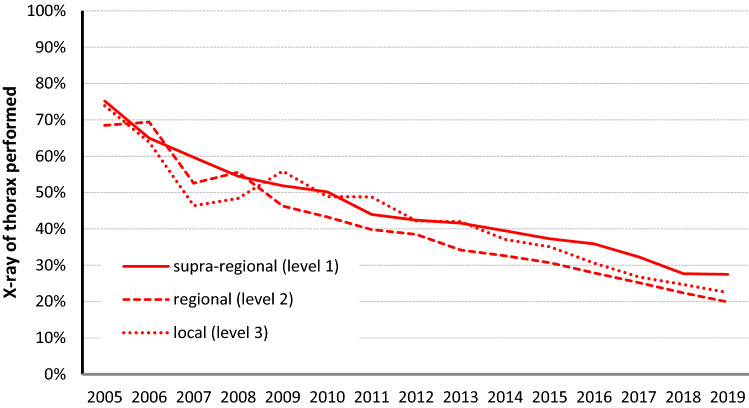
The use of CXR in different designated level of trauma care (data from TR-DGU, Germany 2005–2019, *n* = 251,095 primary admitted patients)

To evaluate the relevance of CXR and the usage of imaging strategy in German trauma centres in the early management of severely injured patients in the TRU over the last 15 years, data from the TR-DGU were acquired. While the use of WBCT increased almost continuously in the years 2005–2010 it reached a stable plateau at about 80% in the following years (Fig. 6). At the same time the usage of CXR decreased continuously over the years (Fig. 7). While almost 75% of the patients had received a CXR in the TRU in 2005, its use exertion decreased to 25% of the cases in 2019. The use of sonography has been high in about 80–90% ever since (Fig. 6). This shift of CXR use over the years occurred parallel in all participating trauma centre levels in Germany (Fig. 7).

## Discussion

Chest radiography (CXR) within the early management of trauma patients plays an important role in the initial evaluation of blunt and penetrating chest trauma, providing rapid imaging information to supplement the history and physical examination, if physicians are well trained to diagnose the pathological findings [3, 4, 9, 13, 27]. An understanding of trauma pathophysiology and related imaging findings for injuries to the chest wall, diaphragm, pleura, lungs, mediastinum, heart, aorta, and great vessels could enable the trauma team to define the right priorities and make rapid decisions [20]. But the presented data showed, that even for this wider range of diagnostic purposes besides the pure mediastinal widening—as investigated in our TR-DGU study—CXR has lost its importance as a first-line diagnostic tool in the TRU. Regardless of their level of care, all Trauma Centers of the German TraumaNetzwerk DGU^®^ have significantly reduced the usage of CXR in the TRU. At the same time the use of eFAST and WBCT has increased continuously, leading to the question if CXR should still be performed within the early trauma management. Even more importantly, if the CXR still is one of the important early imaging tools to define rapid sequence priorities, do the team members of the trauma team still have enough expertise in detecting pathological signs in CXR in the supine position?

As Ho et al. [13] point out, mediastinal widening is only one of multiple radiological signs, which could to be described in the CXR. Regarding MVI, they describe several other signs that should be diagnosed and can lead the attention of the trauma team towards rapid decision making, if diagnosed adequately: (1) surrounding the right pulmonary artery (“ring-around-the-artery”-sign), (2) lateral to the descending aorta (“Naclerio's V”-sign), and (3) superior to the brachiocephalic veins (“V” sign at confluence of brachiocephalic veins) [13]. Mediastinal bleeding or hematoma can result from vascular injury. Large hematomas can produce radiographic irregularity and enlargement of the mediastinum [18, 21]. Proposed criteria for mediastinal widening include a width greater than 8 cm and a mediastinal to chest width ratio greater than 0.25 [27].

The results of this study though demonstrate that an initial CXR in the supine position is not reliable for detecting mediastinal vascular injury through the measurement of mediastinal widening. The sensitivity and specificity when applying different thresholds of maximum M/C ratio are not clinically acceptable. In addition, the aortic contour and hemo- and pneumothorax were not reliably detected in the initial CXR. Although some older publications state that the initial CXR in trauma patients is able to detect a pneumothorax or hemothorax, rib fractures, tracheobronchial injuries, a pneumo-mediastinum, mediastinal haematoma, and lung contusion [12, 18, 21, 27], there is no recent evidence to support these statements [21]. With respect to the diagnosis of mediastinal vascular injuries, different measurements of mediastinal width ratios have been suggested to detect vascular injury. In a retrospective study, Gleeson et al. [9] were able to show that the 8-cm upper limit for normal mediastinal width no longer applied in the modern trauma room. Changes in the position of the X-ray cassette and the lengthening of the distance between the patient and the X-ray source can significantly reduce magnification. They, therefore, suggested a new range of upper limits resulting in maximal normal widths between 8.0 and 10.94 cm [9], though absolute measurements show their limits in anatomical relationships.

**Fig. 8:**
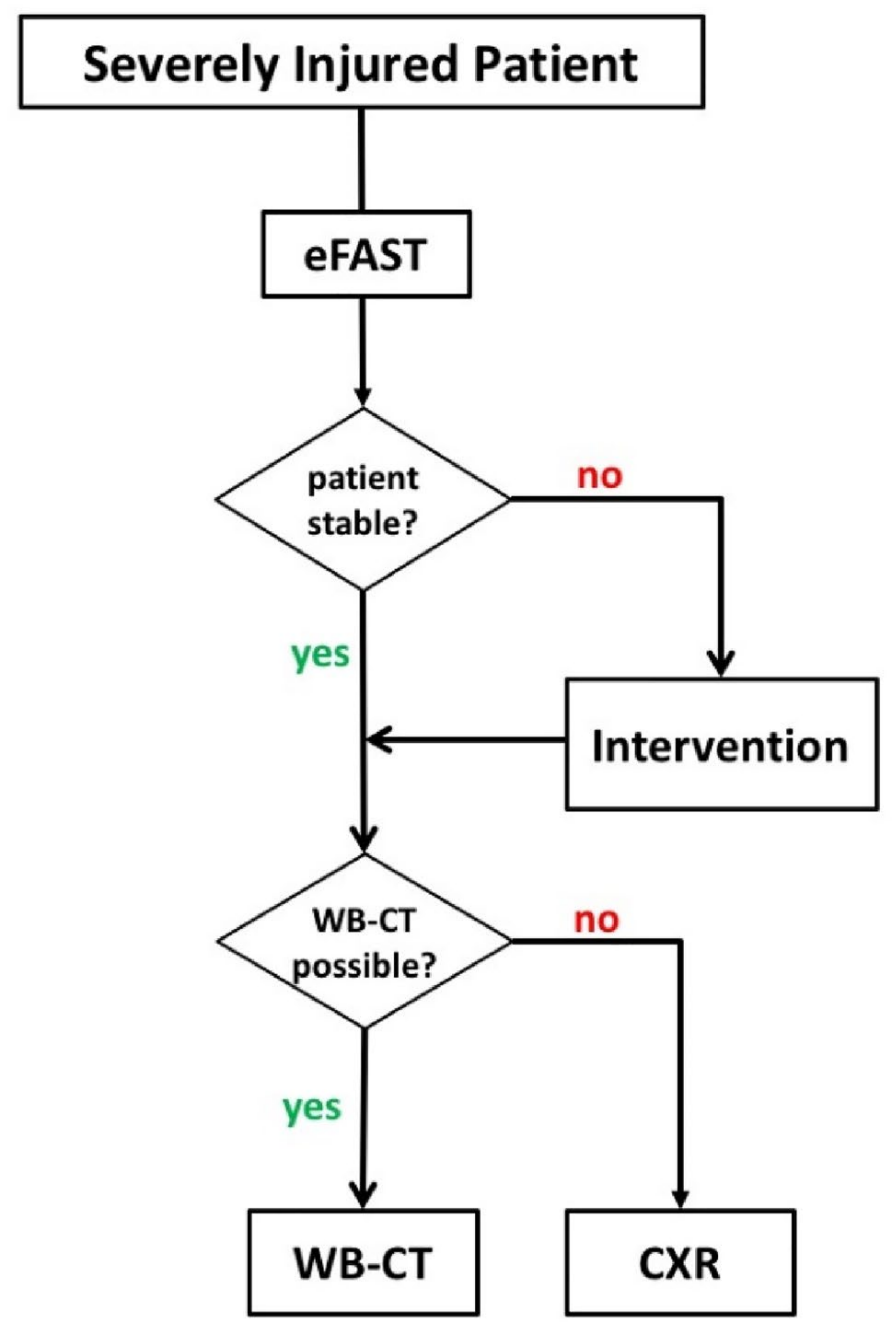
Flow chart for clinical decision-making in the diagnostic pathway during earl trauma management in the TRU, treating cardiorespiratory stable versus unstable patients

Therefore, we state with regard to the presented data, that neither the absolute width nor the M/C ratio in the initial CXR are able to detect MVI in severely injured patients in the TRU and should only be applied in regard to those limits and interpreted carefully. The algorithm being performed in our trauma center is presented in Fig. 8.

Our results support the critical reports on the need and benefit of CXR in the early management of trauma patients in the TRU [12, 23, 25, 26]. Several authors already suggested to omit the initial CXR and to replace it by eFAST [1, 2, 5, 8, 9, 12, 14, 16, 19, 25, 26]. Nevertheless one should not just delete CXR from algorithms in the early trauma management due to the high value in early diagnostic in severe thoracic injury besides pneumothorax, hemothorax, and MVI. Ho et al. [13] have thoroughly shown all different diagnostic findings that are possible to detect in CXR regarding trauma management. The main question that needs to be answered within this early management is, do we expect more important information from the CXR when eFAST has already been performed and WBCT is immediately available in hemodynamically stable patients? If the patient is not stable enough for WBCT and a significant thoracic trauma presumably occurred, CXR is still valuable and recommended (Fig. 8).

Ultrasound of the chest for detecting pneumothorax or hemothorax performed best, with a sensitivity of 100% and a specificity of 94% [25]. A recent publication showed a positive predictive value of the ultrasound of 95% and a negative predictive value of 100% [5]. Another retrospective evaluation in 240 patients showed that ultrasound is equal to CXR in detecting a hemothorax [16]. The sensitivity for both was 96% and specificity was 100%. Limitations in using ultrasound need to be considered if a skin emphysema is present as well as the fact, that the diagnostic value and security of ultrasound is depended on the physician’s skills.

In countries or institutions, where WBCT is not immediately available in every trauma center, it is important to realize that a widened mediastinum (M/C ratio > 0.3 at the aortic arch) can be an indicator of MVI, but it is not a proof. At the same time, a normal M/C ratio does not rule out MVI. Therefore, it is important to consider additional risk factors including the mechanism, severity, and pattern of injury as well as the patient’s physiological status and dynamics. In the literature, the following mechanisms of injury have been identified as causing injuries to the aorta: lateral impact in road traffic accidents [21, 24, 28] and high impact trauma in road traffic accidents (speed > 100 km/h) [6]. Patterns of injury that show a correlation with aortic injuries were multiple rib fractures 1–4 [28] and sternum fractures [11].

## Limitations

While there was no senior radiologist present on the TRU team, communications of CXR findings might have been delayed, although a senior orthopaedic trauma surgeon led the TRU team, which also included an anaesthesiologist and a radiologist. Further limitations are the single-center setting and the retrospective data.

In the presented data, we only focused on the measurement of mediastinal widening as a pathological finding in MVI. The M/C ratio is only one of the clinical pathologies through which the trauma team could be able to detect mediastinal vascular injury early. Since it is the most obvious pathological finding we did not measure other findings out of the CXR.

## Conclusion

Chest radiography still plays an important role in the initial evaluation of chest trauma, providing objective imaging and potentially high diagnostic value. According to the present TRU management guidelines [3, 22], CXR in supine position is performed to detect pneumothorax, hemothorax, and MVI. Our study showed that sensitivity and specificity of CXR in detecting MVI was clinically and statistically not reliable. Previous studies have already shown that CXR was inferior to sonography to detect pneumothorax and hemothorax. Therefore, we challenge the guidelines and suggest that the use of supine CXR in the early management of severely injured patients should be individualized. If sonography and WBCT are available and reasonable for the actual status of the patient, CXR is unnecessary and could delay further more accurate diagnostics. The clinical reality reflected in the usage of CXR and WBCT over time as documented in the TR-DGU seems to support our statement.
